# Artificial Intelligence in Cardiac Amyloidosis: A State-of-the-Art Review

**DOI:** 10.3390/jcm15083037

**Published:** 2026-04-16

**Authors:** Syed Bukhari

**Affiliations:** Division of Cardiology, School of Medicine, Johns Hopkins University, Baltimore, MD 21205, USA; sbukhar3@jh.edu; Tel.: +1-718-674-5378

**Keywords:** cardiac amyloidosis, artificial intelligence, ECG, echocardiogram, cardiac magnetic resonance imaging, nuclear scintigraphy

## Abstract

Cardiac amyloidosis (CA) remains underrecognized due to overlapping features with other cardiovascular conditions, including hypertrophic cardiomyopathy and hypertensive heart disease. Certain ‘red flag’ features across the clinical and imaging spectrum help identify CA. However, these features are often absent, subtle, or inconsistently recognized, particularly in early disease, and are atypical phenotypes. This leads to frequent delays in diagnosis and presentation at advanced stages. Artificial intelligence (AI) offers a promising approach to detect subtle disease signatures by integrating multimodal and longitudinal data beyond human pattern recognition. AI-enhanced electrocardiography has emerged as a scalable screening tool, demonstrating high diagnostic performance and enabling earlier detection. In parallel, echocardiographic AI has evolved toward video-based analysis, improving standardization and reducing inter-reader variability. Similarly, AI applications in cardiac magnetic resonance and nuclear scintigraphy allow for automated quantification and more reproducible assessment of amyloid burden. Beyond diagnosis, emerging models support disease phenotyping, risk stratification, and treatment monitoring. This review synthesizes current applications of AI across multimodal testing in the evaluation and diagnosis of CA.

## 1. Introduction

Cardiac amyloidosis (CA) is caused by myocardial infiltration of amyloid fibrils that are primarily of transthyretin (ATTR-CA) and light-chain (AL-CA) subtypes. CA remains substantially underrecognized in routine practice, largely because its clinical and imaging phenotype overlaps with more prevalent conditions such as heart failure with preserved ejection fraction (HFpEF), hypertensive heart disease, and other causes of left ventricular hypertrophy [1,2,3]. Traditional “red flags” features in CA are neither universally present nor consistently recognized, particularly in early disease or in women and patients with atypical phenotypes [4,5]. As a result, CA is frequently diagnosed late in its natural history, after irreversible myocardial injury has occurred.

AI is uniquely well suited to address these challenges. The diagnostic signal in CA is often subtle, multidimensional, and distributed across modalities, including high-resolution electrocardiography (ECG) waveforms, echocardiographic motion and texture patterns, cardiac magnetic resonance (CMR) tissue characterization, and nuclear scintigraphy tracer uptake. Moreover, CA diagnosis and management are inherently multimodal and longitudinal, requiring integration of imaging, biomarkers, and clinical trajectories over time. Such an analytic task often exceeds the capacity of human pattern recognition alone. Accordingly, contemporary studies increasingly position AI not as an experimental tool, but as a practical strategy to raise early diagnostic suspicion, reduce inter-reader variability, and standardize CA detection across diverse clinical settings.

## 2. Literature Search Strategy and Study Selection

This state-of-the-art narrative review was informed by a structured literature search designed to identify contemporary applications of artificial intelligence (AI) in cardiac amyloidosis (CA). We searched PubMed/MEDLINE and Embase from January 2015 through June 2025 using combinations of the following terms: *cardiac amyloidosis*, *transthyretin*, *light-chain*, *artificial intelligence*, *machine learning*, *deep learning*, *electrocardiography*, *echocardiography*, *cardiac magnetic resonance*, and *nuclear scintigraphy*. Reference lists of key articles were hand-searched to identify additional relevant studies.

Eligible studies included human investigations applying AI or machine learning to ECG, echocardiography, CMR, or nuclear scintigraphy for CA-related diagnostic, prognostic, or monitoring outcomes. We included retrospective and prospective cohort studies and multicenter validation studies. We excluded purely technical or phantom studies without clinical data, editorials without original data, and studies focused on non-cardiac amyloidosis. Given substantial heterogeneity in model architectures, reference standards, outcomes, and reporting practices, formal meta-analysis and quantitative risk-of-bias scoring were not performed. Instead, studies were synthesized qualitatively with emphasis on clinical intent, validation strategy, and translational readiness.

## 3. Machine Learning Approaches for Cardiac Signal and Imaging Analysis

Machine learning approaches for cardiac imaging and signal analysis can be broadly categorized into deep learning, classical machine learning, radiomics-based models, and multimodal fusion frameworks. Supervised deep learning methods—primarily convolutional neural networks and transformer-based architectures—operate directly on raw inputs such as ECG voltage–time signals, echocardiographic video clips, CMR images, and scintigraphy data [6,7,8]. When large, well-annotated datasets are available, these models generally achieve the highest diagnostic performance by capturing complex spatial and temporal patterns. However, this performance advantage comes at the cost of reduced interpretability and greater susceptibility to dataset shift, in addition to substantial data and computational requirements.

In contrast, classical machine learning techniques, including random forests and gradient boosting, rely on engineered features derived from imaging, hemodynamic measurements, or electronic health records. While these approaches are typically more interpretable and easier to implement, they are more dependent on feature selection and may demonstrate lower performance and generalizability compared with deep learning models [9].

Radiomics-based methods occupy an intermediate position, extracting high-dimensional texture features from CMR sequences (e.g., T1 mapping) to quantify myocardial tissue heterogeneity beyond visual assessment. These approaches may offer improved tissue characterization compared with conventional feature-based models, but their performance is highly dependent on image standardization, segmentation quality, and reproducibility across centers [10].

Multimodal fusion models represent an emerging paradigm that integrates complementary data streams—most commonly ECG and echocardiography, with or without clinical variables. Compared with single-modality approaches, these models may improve robustness and diagnostic accuracy by aggregating weak but complementary signals across modalities. However, their implementation is more complex and limited by challenges related to data harmonization, missing data, and clinical integration.

Taken together, these approaches reflect a trade-off between performance, interpretability, scalability, and implementation complexity, and are best viewed as complementary tools tailored to specific clinical use cases.

## 4. ECG AI: Scalable Screening and Opportunistic Detection

**Figure 1:**
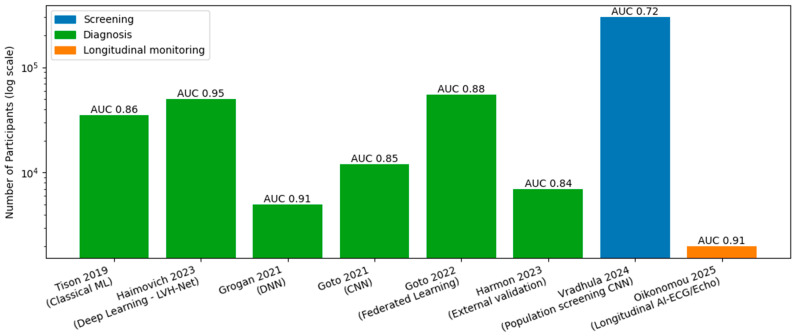
AI-ECG in Cardiac Amyloidosis: Sample Size and Performance in Studies. AI, artificial intelligence; AUC, area under the receiver operating characteristic curve; CNN, convolutional neural network; DL, deep learning; ECG, electrocardiogram; LV, left ventricle; LVH, left ventricular hypertrophy; ML, machine learning; DNN, deep neural network [11,12,13,14,15,16,17,18].

AI-enhanced ECG (AI-ECG) has emerged as one of the most scalable and pragmatic entry points for early detection of CA [11,12,13,14,15,16,17,18] (Figure 1). Deep learning models trained on standard 12-lead ECGs can identify CA-associated electrical signatures even when routine clinical interpretation is non-diagnostic and classic red flags—such as low voltage or pseudoinfarction patterns—are absent. This is particularly relevant in early disease, ATTR-CA, and in patient subgroups with competing causes of left ventricular hypertrophy, where ECG findings are often subtle or nonspecific. Because ECGs are inexpensive, ubiquitous, and already embedded in routine clinical workflows, AI-ECG approaches are well suited for opportunistic screening and automated triage to downstream confirmatory testing.

Several single- and multicenter studies have demonstrated strong diagnostic discrimination of AI-ECG models for CA, including both ATTR and AL subtypes. In a large retrospective cohort of 4995 individuals (2541 with AL-CA or ATTR-CA and 2454 matched controls), a deep learning model trained on standard 12-lead ECGs identified CA with high accuracy (AUC 0.91), substantially outperformed conventional ECG criteria, and detected disease more than six months prior to clinical diagnosis in a majority of cases [13]. In another study, using ECG and machine learning, detailed electroanatomical mapping in CA revealed characteristic ventricular voltage and activation abnormalities that were translatable to simple 12-lead ECG patterns, enabling markedly improved diagnostic performance after focused training (AUC increasing from 0.69 to 0.97) without reliance on advanced imaging [19]. Finally, in a meta-analysis of five studies comprising seven derivation and validation cohorts (8639 derivation and 3843 validation participants), AI-enhanced ECG models demonstrated high pooled diagnostic performance for CA overall (AUC 0.89, 95% CI 0.86–0.91), with similarly strong discrimination for ATTR-CA (AUC 0.90, 95% CI 0.86–0.95) and more modest performance for AL-CA (AUC 0.80, 95% CI 0.80–0.93) [20]. These findings underscore both the promise of AI-ECG as a screening tool and the importance of local validation before deployment.

While individual study design, labeling strategy, and reference standards varied, the consistency of discrimination across cohorts supports the feasibility of AI-ECG for screening-style workflows, particularly as a rule-out or enrichment tool rather than a standalone diagnostic test. Importantly, most studies emphasize probabilistic risk outputs rather than binary classification, aligning with real-world triage and shared decision-making paradigms.

From a practical standpoint, several implementation use cases have been proposed. One approach involves EHR-integrated, background execution of AI-ECG models on all incoming ECGs, generating automated alerts for patients exceeding a predefined risk threshold and prompting targeted chart review and stepwise evaluation [21]. Another use case is pre-test enrichment, in which AI-ECG is used to selectively identify patients who may benefit most from echocardiography, CMR, or bone-avid scintigraphy, thereby improving diagnostic yield and reducing unnecessary downstream testing [12]. These strategies are particularly appealing in health systems with high ECG volumes and limited access to advanced imaging resources.

Despite these advantages, important limitations remain. Dataset shift—driven by differences in ECG acquisition hardware, filtering, lead placement conventions, and the prevalence of conduction disease or paced rhythms—can meaningfully degrade model performance outside the development environment [22]. Label noise is another major challenge, as “ground truth” CA definitions vary across studies and may rely on biopsy, imaging-based criteria, or clinician diagnosis, each with distinct sensitivities and biases [23,24]. Finally, workflow risk is nontrivial: highly sensitive AI-ECG tools can overwhelm clinics with false positives if not paired with clearly defined downstream testing pathways, referral thresholds, and capacity planning [25]. Accordingly, successful clinical deployment requires careful integration with multidisciplinary amyloidosis care pathways rather than isolated algorithmic implementation.

## 5. Echocardiography AI: From Engineered Features to Video-Level Detection

Echocardiography has long been central to the evaluation of CA, providing key structural and functional clues such as increased wall thickness, restrictive filling patterns, atrial enlargement, and characteristic abnormalities in longitudinal strain [26]. However, recognition of CA on echocardiography remains highly dependent on reader expertise, clinical suspicion, and image quality, leading to substantial inter-reader variability—particularly in early disease, atypical phenotypes, and patients with competing causes of left ventricular hypertrophy [27,28]. Even well-described markers such as relative apical sparing may be absent or subtle, limiting sensitivity in routine practice [29]. Against this backdrop, AI offers a means to standardize interpretation, surface latent patterns, and reduce reliance on subjective visual recognition.

**Figure 2:**
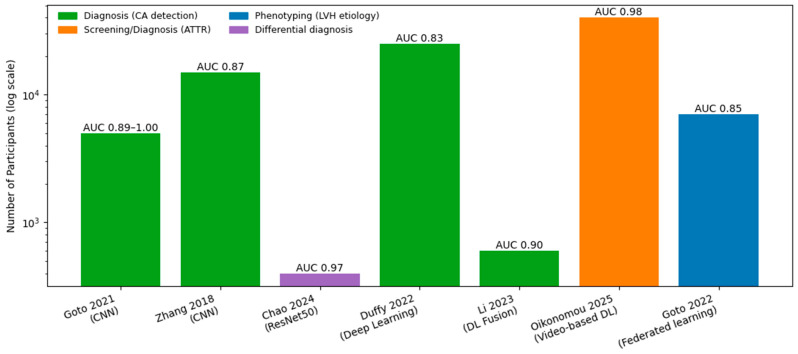
AI-echocardiography in Cardiac Amyloidosis: Sample Size and Performance in Studies. AI, artificial intelligence; AUC, area under the receiver operating characteristic curve; CA, cardiac amyloidosis; ATTR, transthyretin amyloidosis; CNN, convolutional neural network; DL, deep learning; ECG, electrocardiogram; LV, left ventricle; LVH, left ventricular hypertrophy; ML, machine learning [14,16,18,32,33,34,35].

Broadly, two complementary AI approaches have emerged in echocardiography for CA detection [13,18,30,31,32,33,34,35] (Figure 2). The first involves feature-based machine learning models that leverage conventional echocardiographic measurements, including wall thickness, Doppler indices, left atrial size, and strain-derived parameters [33]. These models often use structured data already stored in echocardiography reporting systems or electronic health records, making them relatively easy to implement and explain. Several studies have demonstrated good diagnostic performance using such approaches, particularly when strain metrics are included, although performance remains sensitive to measurement quality, vendor-specific differences, and local acquisition protocols. In a cohort of 51 patients with confirmed CA, fully automated AI-derived LVEF and GLS from apical 2- and 4-chamber echocardiographic views showed strong agreement with manual measurements (r ≈ 0.74–0.83), with sensitivities of 70–100% and specificities of 67–86% for detecting abnormal function, while substantially reducing measurement time [31]. Nonetheless, feature-based models provide an important bridge between traditional echocardiography and AI-driven augmentation, offering interpretability and lower barriers to deployment.

More recently, the field has shifted toward video-based deep learning approaches that train directly on raw echocardiographic image sequences, most commonly apical four-chamber clips, with or without additional views [13,32,36]. By learning directly from pixel-level motion and texture patterns, these models aim to capture disease-specific signatures—such as subtle abnormalities in myocardial thickening, relaxation, and speckle texture—that are not fully represented by derived summary metrics. In a multicenter cohort of 23,745 patients, a fully automated deep learning echocardiography workflow accurately quantified left ventricular wall thickness (mean absolute error ~1–2 mm) and distinguished CA (AUC 0.79–0.83) and hypertrophic cardiomyopathy (AUC 0.89–0.98), addressing key limitations of human measurement variability and diagnostic overlap [32]. This shift from “feature engineering” to end-to-end video learning represents a major conceptual advance in echo-based CA detection.

Building on modality-specific advances, multimodal pipelines that integrate ECG and echocardiography have further strengthened performance and generalizability. A landmark Multicenter AI models using ECG and echocardiography detected CA with high discrimination (C-statistics up to 0.91 and 1.00, respectively), with ECG pre-screening improving echocardiographic PPV from 33% to 74–77% at 67% recall [13]. This work illustrated how complementary signals across modalities can be fused to improve robustness and reduce false positives, reinforcing the concept that CA is best detected through integrated, multimodal analysis rather than reliance on any single test.

Looking ahead, several trajectories are likely to shape the next phase of echocardiography AI in CA. Automated strain computation and image quality control are expected to reduce technical variability and expand access to advanced deformation analysis [37]. Opportunistic CA screening from routine echocardiograms performed for heart failure, murmurs, left ventricular hypertrophy, or aortic stenosis represents a particularly attractive use case, given the high pretest probability in these populations. Finally, longitudinal AI-driven quantification may enable more consistent assessment of disease progression and therapeutic response, reducing inter-reader variability and supporting standardized monitoring in both clinical practice and trials. Together, these developments position echocardiography AI as a central component of scalable, multimodal CA detection strategies.

## 6. CMR AI: Tissue Characterization, Radiomics, and Automated Quantification

CMR occupies a unique position in the evaluation of CA, offering unparalleled myocardial tissue characterization through late gadolinium enhancement (LGE), native T1 mapping, and extracellular volume (ECV) quantification [38,39]. These techniques enable detection of diffuse infiltration, disease staging, and prognostication across amyloid subtypes. However, CMR is time-intensive, technically demanding, and dependent on specialized expertise, which limits scalability and contributes to inter-site variability in acquisition and interpretation. AI applications in CMR are therefore focused not only on improving diagnostic performance, but also on reducing technical barriers and standardizing analysis to support broader and more consistent use in CA care.

One major axis of CMR-AI development involves workflow automation and standardization. Automated segmentation of cardiac chambers and myocardial regions, along with automated generation of parametric maps, has been shown to substantially reduce analysis time and inter-observer variability [8,40]. In AL-CA, AI-driven segmentation pipelines have been used to derive native T1 and ECV measures with diagnostic and prognostic relevance comparable to expert manual analysis, supporting both risk stratification and longitudinal follow-up [41]. More broadly, the radiology literature has demonstrated AI applications across the CMR pipeline—including accelerated image acquisition, reconstruction, motion correction, segmentation, and quantitative mapping—which is particularly relevant for CA protocols that are often lengthy and resource-intensive. These advances position AI as a key enabler of reproducible, high-throughput CMR assessment in both referral centers and emerging amyloidosis programs.

A second, complementary line of investigation centers on radiomics and machine learning applied to CMR for CA detection and phenotyping [42]. Radiomics approaches extract high-dimensional quantitative features from LGE images—such as texture, signal heterogeneity, and spatial distribution—that extend beyond what is readily appreciated by visual inspection [10]. Several studies have demonstrated that radiomics-based models can distinguish CA from other causes of left ventricular hypertrophy, including hypertrophic cardiomyopathy and hypertensive heart disease, with promising diagnostic performance [43,44]. Importantly, these methods aim not merely to replicate expert interpretation, but to uncover latent tissue signatures that may reflect amyloid burden, infiltration patterns, or disease stage, raising the possibility of more granular phenotyping and risk assessment.

Despite these advances, key gaps remain before CMR-AI can be widely deployed in CA. Generalizability across vendors, field strengths, and pulse sequences remains a major challenge, as CMR acquisition parameters vary substantially across centers and can strongly influence quantitative outputs. Harmonization of labels is another unresolved issue, as many datasets combine ATTR-CA and AL-CA, span wide ranges of disease severity, or include patients at different treatment stages, complicating model interpretation and clinical translation. Finally, most studies to date have been retrospective and performed in highly selected populations; prospective validation in real-world “left ventricular hypertrophy clinics”—where the differential diagnosis is broad and pretest probability of CA is modest—will be essential to define clinical utility and cost-effectiveness. Addressing these gaps will determine whether CMR-AI evolves from a powerful research tool into a scalable component of routine CA evaluation.

## 7. Nuclear Scintigraphy AI: Automated Detection and Quantitative Assessment

Bone-avid radiotracer scintigraphy using agents such as 99mTc–pyrophosphate (PYP), 99mTc–DPD, or 99mTc–HMDP has become central to the noninvasive diagnosis of ATTR-CA when interpreted in the appropriate clinical context [45]. Despite its high diagnostic specificity, real-world interpretation remains vulnerable to variability in visual grading, region-of-interest (ROI) placement, planar versus SPECT assessment, and reader experience—factors that can influence both diagnostic classification and longitudinal assessment. AI applications in nuclear cardiology are therefore well aligned with the core pain points of scintigraphy: standardization, automation, and reproducible quantification.

**Figure 3:**
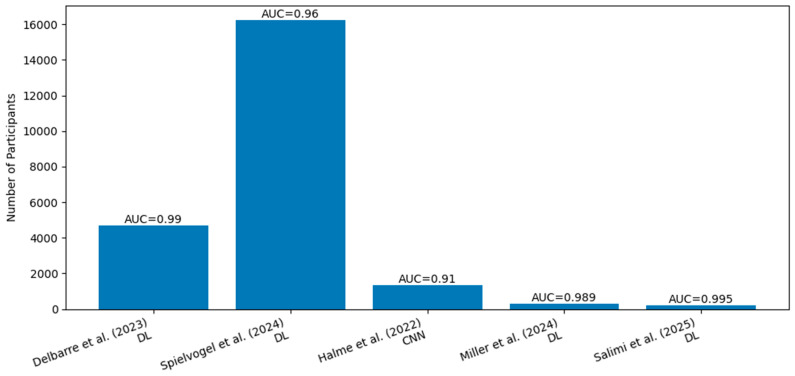
AI-Nuclear scintigraphy in Cardiac Amyloidosis: Sample Size and Performance in Studies. AI, artificial intelligence; AUC, area under the receiver operating characteristic curve; DL, deep learning; CNN, convolutional neural network [46,48,49,50,51].

Recent advances have focused on deep learning-based quantification of cardiac radiotracer uptake, with automated myocardial segmentation enabling fully automated calculation of volumetric and intensity-based uptake metrics from SPECT or planar imaging [46,47,48,49,50,51] (Figure 3). Studies using AI-driven quantification of 99mTc–PYP uptake have demonstrated close agreement with expert-defined measurements while substantially reducing operator dependence, suggesting a pathway toward more reproducible classification and follow-up assessment. Importantly, such approaches move beyond traditional semi-quantitative heart-to-contralateral (H/CL) ratios toward more granular, three-dimensional characterization of tracer distribution, which may better reflect amyloid burden.

A parallel and increasingly compelling application involves opportunistic detection of ATTR-CA on routine scintigraphy performed for non-cardiac indications. Deep learning models trained to identify amyloid-suggestive cardiac uptake on whole-body or regional scans—originally acquired for oncologic or skeletal evaluation—have been reported, highlighting the potential to surface previously unrecognized ATTR-CA without additional imaging or radiation exposure. This screening paradigm aligns with broader trends in opportunistic imaging analytics and is particularly attractive given the aging population undergoing nuclear studies for unrelated conditions. By standardizing detection and flagging suspicious studies for confirmatory evaluation, AI could meaningfully advance earlier case finding.

More recently, investigators have extended deep learning approaches to total-body scintigraphy across different bone tracers and scanner platforms, reflecting a deliberate move toward vendor- and tracer-agnostic deployment. Models trained and validated across multiple tracers (PYP, DPD, HMDP) and institutions suggest that AI may help harmonize interpretation across international practice patterns, where tracer choice and acquisition protocols vary widely. Such generalizability is especially important for ATTR-CA, where diagnostic pathways differ substantially between North America and Europe.

From a practical standpoint, nuclear scintigraphy AI offers several advantages that directly address clinical needs. Automated analysis promises more consistent reads across sites and readers, reducing variability in both diagnosis and follow-up. Improved quantitative measures may support longitudinal monitoring of disease burden and therapeutic response as disease-modifying treatments expand. Finally, opportunistic detection from non-cardiac nuclear studies represents a low-cost, high-yield strategy to identify patients earlier in the disease course. Together, these developments position AI-enhanced scintigraphy as a natural complement to ECG-, echo-, and CMR-based approaches in a comprehensive, multimodal CA detection ecosystem.

## 8. Beyond Diagnosis: Prognosis, Phenotyping, and Treatment Monitoring

As AI-based tools for CA mature, their role is increasingly framed not merely as diagnostic classifiers, but as platforms for disease phenotyping, risk stratification, and longitudinal monitoring. Rather than asking only whether CA is present, contemporary models aim to estimate the likelihood of ATTR-CA versus AL-CA, infer disease stage or burden using imaging and electrical proxies, and predict clinically meaningful outcomes such as heart failure hospitalization, arrhythmias, and mortality. These approaches typically leverage multimodal inputs—including ECG embeddings, echocardiographic deformation patterns, CMR tissue metrics, and scintigraphic quantification—to capture complementary aspects of myocardial involvement that are difficult to integrate manually.

Studies suggest that AI-derived features may carry prognostic information beyond traditional clinical and imaging markers. For example, latent ECG representations and automated strain or mapping metrics have been associated with adverse outcomes in CA cohorts, raising the possibility of AI-assisted risk stratification and treatment prioritization [52]. This highlights the prognostic dimension as a key frontier for the field. However, most prognostic models remain retrospective and require careful recalibration in the contemporary treatment era, as disease-modifying therapies such as tafamidis and emerging AL-directed treatments fundamentally alter baseline risk and disease trajectories.

AI is also increasingly being applied to longitudinal monitoring of disease progression and treatment response. Automated and reproducible quantification of strain patterns, ECG embeddings, parametric mapping values (native T1, ECV), and scintigraphic uptake may enable more sensitive detection of subtle changes over time compared with conventional approaches. This is particularly relevant as therapies are initiated earlier, when absolute changes are small but clinically meaningful. Standardized AI-driven metrics may further facilitate harmonized endpoints in clinical trials and registries by reducing inter-reader variability.

## 9. Implementation: Translating AI into Clinical Workflows

Successful clinical deployment of AI in CA depends on integration into existing workflows rather than standalone algorithmic outputs. A pragmatic model begins with opportunistic screening, typically using ECG or echocardiography, to identify patients at elevated risk, followed by clinical adjudication incorporating heart failure phenotype, left ventricular hypertrophy context, red flags, and monoclonal protein testing. Confirmatory evaluation with CMR or bone-tracer scintigraphy is then pursued, with biopsy reserved for selected cases. Importantly, such pathways should include feedback mechanisms to track false positives and negatives, enable local recalibration, and monitor model performance over time.

Traditional performance metrics such as AUC, while useful, are insufficient to determine real-world utility. Calibration, decision-curve analysis, and net benefit across clinically relevant thresholds better reflect clinical value. In addition, fairness across demographic subgroups and operational metrics, such as referral volume, diagnostic yield, and time-to-diagnosis, are essential to ensure that AI improves care rather than simply redistributes workload.

## 10. Key Limitations and Unresolved Challenges

Despite promising advances, several challenges constrain clinical translation. Ground-truth uncertainty remains significant due to heterogeneity in diagnostic standards, including biopsy, imaging-based criteria, and clinician adjudication. Spectrum bias is common, as many models are trained on enriched cohorts with advanced disease, limiting generalizability to real-world populations such as HFpEF or mild hypertrophy cohorts. Dataset shift across acquisition protocols, vendors, and patient populations further complicates deployment.

Explainability also remains central to clinician trust, with increasing emphasis on interpretable outputs such as saliency mapping and feature-based models. Finally, robust prospective validation is needed to demonstrate that AI meaningfully improves outcomes, including earlier diagnosis, improved risk stratification, and better patient-centered endpoints.

## 11. Future Directions: From Detection to Clinical Impact

The field is now transitioning from proof-of-concept detection toward demonstrating clinical value. Priority areas include prospective, pragmatic trials embedded within real-world care pathways to evaluate whether AI shortens time-to-diagnosis, improves diagnostic yield, and facilitates earlier initiation of therapy. As treatment paradigms evolve, models will require recalibration to reflect changing disease trajectories.

More broadly, future efforts should focus on defining where and how AI adds value—identifying the right patients, at the right time, within the diagnostic pathway. Multimodal foundation models integrating ECG, echocardiography, CMR, scintigraphy, and clinical data are particularly promising, given the inherently multimodal nature of CA. Ultimately, successful implementation will depend on embedding AI within workflow-aware, multidisciplinary care pathways, positioning it as a longitudinal decision-support tool rather than a standalone diagnostic solution.

## 12. Conclusions

AI is rapidly transforming the detection and evaluation of CA by enabling earlier, more consistent recognition of disease signals that are often subtle, multimodal, and missed by conventional interpretation. While diagnostic performance across ECG, echocardiography, CMR, and nuclear imaging is promising, real-world impact will depend on rigorous external validation, calibration, and integration into clinically actionable care pathways. Ultimately, AI’s greatest value in CA may lie not as a standalone diagnostic tool, but as an embedded, longitudinal decision-support system that improves timeliness of diagnosis, risk stratification, and treatment monitoring in the modern therapeutic era.

## Data Availability

No new data were created or analyzed in this study.
